# Akzeptanz digitaler Apps in der Nachsorge von Patient*innen mit Kopf-Hals-Tumoren

**DOI:** 10.1007/s00106-024-01463-6

**Published:** 2024-05-03

**Authors:** Christopher Weusthof, Zhaojun Zhu, Anne Ruck, Amir Bolooki, Oliver Schöffski, Barbara Wollenberg, Markus Wirth

**Affiliations:** 1 Klinik und Poliklinik für Hals-Nasen-Ohrenheilkunde, Klinikum rechts der Isar, Technische Universität München, Ismaninger Straße 22, 81675 München, Deutschland; 2 Lehrstuhl für Gesundheitsmanagement, Universität Erlangen-Nürnberg, Nürnberg, Deutschland

**Keywords:** Fragebogen, Datenerfassung, „Patient-reported outcome measures“, Lebensqualität, Humanes Papillomvirus, Questionnaire, Data collection, Patient-reported outcome measures, Quality of life, Human papilloma virus

## Abstract

**Hintergrund:**

„Patient-reported outcome measures“ führen bei Tumorpatient*innen sowohl zu einer verbesserten Lebensqualität als auch möglicherweise zu einer früheren Erkennung von Tumorrezidiven.

**Fragestellung:**

Erfragt wurde das Interesse der Patient*innen an einer Ergänzung der Tumornachsorge durch Apps zur Erfassung der Lebensqualität (patient-reported outcome [PRO]).

**Material und Methode:**

Dazu erfolgte die Auswertung eines selbstständig erstellten Fragebogens zur Erfassung des Interesses von Tumorpatient*innen an einer digitalisierten Form der Tumornachsorge (*n* = 110)

**Ergebnisse:**

In der Studie zeigten sich Tumorpatient*innen zum Großteil interessiert an einer App-Nutzung zur Tumorerkrankung. Unter Einbeziehung des Alters stieg die Anzahl erwartungsgemäß bei Patient*innen < 60 Jahren noch weiter. Dazu passend zeigten für humanes Papillomavirus (HPV-)positive Patient*innen ein signifikant größeres Interesse (*p* = 0,021).

**Schlussfolgerung:**

Die Einführung einer App wird vom Großteil der Tumorpatient*innen befürwortet. Neben der Erfassung des PRO könnten hier weitere Anwendungen (Terminerinnerung, Patientenakte, Sport‑/Ernährungsprogramme) integriert werden.

**Zusatzmaterial online:**

Die Online-Version dieses Beitrags (10.1007/s00106-024-01463-6) enthält den Fragebogen zur Erfassung des Interesses von Tumorpatient*innen an einer digitalisierten Form der Tumornachsorge.

## Abstract (en)

**Background:**

Patient-reported outcome (PRO) measures lead to improved quality of life in cancer patients and possibly to earlier detection of tumor recurrence.

**Objective:**

Patient interest in complementing tumor follow-up care with apps for recording PROs was assessed using a questionnaire.

**Materials and methods:**

An independently created questionnaire to record the interest of tumor patients in a digitalized form of tumor follow-up care was evaluated (*n* = 110).

**Results:**

In the study, the majority of tumor patients were interested in using an app for tumor disease. Taking age into account, the number increased even further for patients < 60 years of age, as expected. In line with this, human papillomavirus (HPV)-positive patients showed significantly greater interest (*p* = 0.021).

**Conclusion:**

The introduction of an app is supported by the majority of tumor patients. In addition to recording PROs, other applications (appointment reminders, patient files, sports/nutrition programs) could also be integrated.

Eine strukturierte onkologische Nachsorge gilt als wichtigster Baustein in der Versorgung von Tumorpatient*innen nach Therapieabschluss. Hierbei geht es v. a. um die frühzeitige Erkennung von Tumorrezidiven, Fernmetastasierung und metachronen Zweitkarzinomen sowie um die Behandlung von therapieassoziierten Nebenwirkungen. Bisher konnte bei zumeist symptomatischen Rezidiven durch die onkologische Nachsorge kein signifikant besseres Überleben, aber zumindest eine Verbesserung der Lebensqualität gezeigt werden [1]. Hierin könnte die Stärke einer Symptomabfrage über eine entsprechende App auf dem Smartphone als Ergänzung im Sinne einer digitalen Tumornachsorge liegen.

## Onkologische Nachsorge in der HNO-Heilkunde

Trotz der multimodalen Therapiekonzepte kommt es in etwa 25 % der Kopf-Hals-Tumoren zu einem lokoregionären Rezidiv [2, 3]. Die Auftretenswahrscheinlichkeit eines Rezidivs ist innerhalb der ersten 2 Jahre nach Erstdiagnose am höchsten [4]. Dabei ist das Tumorstadium bei Erstdiagnose entscheidend für die weitere Prognose. Bei lokal fortgeschrittenen Tumoren (Stadium III–IV) liegt die Rezidivrate in diesem Zeitraum bei etwa 20 %, wohingegen die Rate in frühen Stadien unter 10 % beträgt [5].

Zusätzlich zum initialen Tumorstadium zählt insbesondere die R1- bzw. R2-Resektion während der chirurgischen Therapie als Faktor für die Wahrscheinlichkeit eines Rezidivs. Hier erweist sich das rezidivfreie Überleben als um 40 % (R1) bzw. 60 % (R2) reduziert [6]. Weitere Hauptrisikofaktoren für das Auftreten eines Tumorrezidivs sind ein extrakapsuläres Wachstum (ECE+), eine perineurale Invasion, ein pN-Status ≥ N2 sowie ein fortgesetzter Alkohol- und Nikotinabusus [7–9].

Aufgrund mangelnder Evidenz aus prospektiven und randomisierten Studien fehlt bis heute eine international einheitliche Leitlinie zu den Untersuchungsabständen und der Durchführung von Kontrollbildgebungen im Rahmen der Nachsorge von Patient*innen mit Kopf-Hals-Karzinomen. In der Studie von Szturz et al. konnte gezeigt werden, dass die Nachsorge nicht zu einem verlängerten Überleben, aber zu einer Steigerung der Lebensqualität führt [1]. Ein Grund hierfür könnte sein, dass 56–85 % der Rezidive symptomatisch sind und wahrscheinlich auch bei einer symptombasierten Wiedervorstellung entdeckt worden wären [1, 10, 11].

Bei der Intensität der Nachsorge sollte daher auch die emotionale und finanzielle Belastung der Patient*innen durch Untersuchungen sowie körperliche Umstände durch weite Anreisen an die jeweiligen Tumorzentren berücksichtigt werden [12]. Daher etablierten die Kolleg*innen der Klinik für Hals-Nasen-Ohrenheilkunde des Universitätsklinikums Ulm im Rahmen der „NachO-Studie“ das sog. Ulmer Modell, in dem sich die Patient*innen im Studienarm wechselweise im Tumorzentrum und bei der/beim heimatnahen niedergelassenen HNO-Fachärzt*in zur klinischen Nachsorgeuntersuchung vorstellten [13]. Im Anschluss an die heimatnahe Vorstellung erfolgte stets eine telefonische Rücksprache. Laut vorläufigen Ergebnissen zeige sich eine erhöhte Compliance zur Teilnahme an den Nachsorgeuntersuchungen unter den Patient*innen im Studienarm. Außerdem sei die ärztliche Versorgung durch die stärkere Einbeziehung der heimatnahen HNO-Fachärzt*innen verbessert [13].

Da die Nachsorge das Überleben nicht verbessert, ist insbesondere die Behandlung der therapieassoziierten Nebenwirkungen und die Vermeidung von Komplikationen Aufgabe der Nachsorge. Nebenwirkungen nach einer Radiochemotherapie, die z. B. das Schlucken betreffen und mit Schmerzen, Gewichtsverlust, Mundtrockenheit und Zahnproblemen einhergehen, kommen z. T. erst nach Jahren zum Vorschein. Vor allem im Bereich des Pharynx und Larynx war eine relevante Rate von 43 % Spättoxizitätsfolgen nachweisbar [14]. Diese können zeiteffizient mittels „patient-reported outcome measures“ (PROM) in der Tumornachsorge erhoben werden.

## „Patient-reported outcome measures“

Neben den beschriebenen objektiven Parametern ist das Befinden und insbesondere die Lebensqualität („patient-reported outcome“, PRO) während und nach der Therapie für Patient*innen entscheidend. Um die Behandlung zu verbessern, ist es essenziell, individuell auf die Bedürfnisse und Beschwerden der Patient*innen einzugehen [15]. Therapieassoziierte Nebenwirkungen bzw. der Einfluss der Tumorerkrankung auf Symptome, Körperfunktionen sowie die krankheitsbezogene Lebensqualität können mittels PROM objektiviert werden [15]. Diese werden im Rahmen von klinischen Studien i. d. R. durch Fragebögen erhoben, um die Krankheitslast und Effektivität von Interventionen aus Patient*innensicht zu eruieren [15]. Im Rahmen der alltäglichen Behandlung von individuellen Patient*innen ist die Erhebung von PROM allerdings noch relativ neu, bietet aber die Möglichkeit einer individuellen Versorgung passend zu den jeweiligen Bedürfnissen.

## Digitale Erfassung von PROM

Aufgrund des komplizierten Handlings der papierbasierten Erhebung von PROM (mangelhafte Lagerung, manuelle Auswertung, Unübersichtlichkeit) stehen mittlerweile verschiedene elektronische Systeme zur Erfassung der PRO („electronic patient-reported outcome“, ePRO) für Tumorerkrankungen zur Verfügung [15, 16]. Unterschieden werden hierbei behandlungszentrierte Systeme während der Therapie und patient*innenzentrierte Systeme, die während der Behandlung und der Nachsorge eingesetzt werden [16]. Bereits 2014 wurden in einem Review 33 Systeme v. a. aus den USA gelistet, wohingegen nur das Computer-based Health Evaluation System (CHES) von der Universität Innsbruck aus dem deutschsprachigen Raum stammte [16].

Im Zeitalter der Digitalisierung ergibt sich im Rahmen der onkologischen Nachsorge zwangsläufig die Frage, inwiefern die Erfassung von PRO und die Nachsorge durch eine App komplementiert werden könnte. Insgesamt erscheint das Potenzial der Nutzung einer App ergänzend zur Tumorbehandlung und -nachsorge groß. Bislang ist allerdings noch unklar, inwieweit die zum Großteil älteren Patient*innen digital unterstützte Systeme auch annehmen werden und ob hierbei der Status in Bezug auf das humane Papillomavirus (HPV) eine Rolle spielt.

## Material und Methoden

Um dieses Thema zu untersuchen, erstellten die Autoren einen Fragebogen in deutscher Sprache mit insgesamt 20 Fragen („multiple choice“ von 1–5, Mehrfachauswahl, Freitext), der in einem Zeitraum von 5 Monaten an insgesamt 110 Patient*innen aus der Klinik und Poliklinik für Hals-Nasen-Ohrenheilkunde des Klinikums rechts der Isar der TU München ausgeteilt wurde. Die Befragung wurde im Rahmen der Masterarbeit von M. W. zum Master of Public Health (MHBA) konzipiert, und angelehnt an die Befragung von Wald et al. {Wald, 2022 #1245} [18] wurde jedoch ein stärkerer Fokus auf Erfordernisse einer Smartphone-App gelegt und zusätzlich der HPV-Status abgefragt. Der Fragebogen wurde bisher nicht im Rahmen einer großen, repräsentativen Stichprobe validiert. Die inkludierten Fragen wurden explizit ausgewählt, um einerseits allgemeine Informationen zu generieren und das Interesse an verschiedenen Facetten einer möglichen App-Nutzung zu erfassen. Eingeschlossen wurden Tumorpatient*innen aller Kopf-Hals-Tumoren (neben den in Tab. 1 einzeln aufgeführten Lokalisationen außerdem Nasen‑, Nasennebenhöhlen‑, Speicheldrüsenkarzinome sowie maligne Hauttumoren im Kopf-Hals-Bereich) im Rahmen der onkologischen Nachsorge sowie während der Therapie. Hierbei wurden die Patient*innen vor der Befragung ausführlich über den Sinn des Fragebogens und die kurz- und langfristigen Ziele aufgeklärt. Insgesamt 8 Fragebögen mussten von der Auswertung ausgeschlossen werden, da die Patient*innen die Einwilligungserklärung zur anonymen Verarbeitung der Daten nicht unterschrieben.

**Tab. 1:** Kreuztabelle zu unterschiedlichen Einflussfaktoren auf das Interesse an einer App-Nutzung im Rahmen der Tumornachsorge

		Kein Interesse App-Nutzung		Interesse App-Nutzung		
Merkmal	Kategorie	*n*	**%**	*n*	**%**	*p*-Wert^a^
*Alter (Jahre)*	< 60	9	9,4	37	38,5	**0,0471**
	≥ 60	19	19,8	31	32,3	–
*Geschlecht*	Männlich	18	18,0	47	47,0	0,493
	Weiblich	12	12,0	23	23,0	–
*Tumorursprung*	Mundhöhle	4	4,2	9	9,5	0,345
	Oropharynx	4	4,2	20	21,1	–
	Hypopharynx	1	1,1	7	7,4	–
	Larynx	7	7,4	10	10,5	–
	Andere	7	7,4	26	27,4	–
*HPV*	Nein	7	17,9	18	46,2	0,745
	Ja	1	2,6	13	33,3	–
*Schulabschluss*	< Abitur	20	21,5	39	41,9	0,122
	≥ Abitur	8	8,6	26	28,0	–
*Behandlung*	Therapie	3	3,1	10	10,3	0,621
	Nachsorge	25	25,8	59	60,8	–
*Information*	Nicht ausreichend	9	10,8	6	7,2	**0,0053**
	Ausreichend	16	19,3	52	62,7	–
*Zusätzliche*	Nein	17	21,5	19	24,1	**0,00014**
*Information*	Ja	4	5,1	39	49,4	–
*Smartphone*	Nein	11	11,1	0	0,0	**<** **0,00001**
	Ja	19	19,2	69	69,7	–
*Betriebssystem*	IOS	6	7,3	29	35,4	0,816
	Android	9	11,0	38	46,3	–

^a^*χ*^*2*^*-Test; p‑Werte* *<* *0,05 sind fett markiert*

Zur Erstellung des Violinen-Plots wurde R Version 4.0.3 (Posit PBC, Boston, MA, USA) mit den zugehörigen packages „ggplot2“, „ggpubr“, „tidyverse“ und „readr“ verwendet. Die Berechnung der Signifikanz erfolgte über einen t‑Test.

Zur Berechnung der Signifikanz der von den Autor*innen erstellten Kreuztabellen wurden *χ*^*2*^-Tests in RStudio (Posit PBC, Boston, MA, USA), Version 4.0.3, durchgeführt. Das Balken- sowie das Säulendiagramm wurde mithilfe von Microsoft Excel (Microsoft, Redmond, WA, USA) erstellt. Zur besseren Beurteilung wurden die Antworten, die keiner Gruppe (Interesse vs. kein Interesse) definitiv zugeordnet werden konnten, aus diesen Analysen ausgeschlossen oder explizit aufgeführt.

## Ergebnisse

**Abb. 1:**
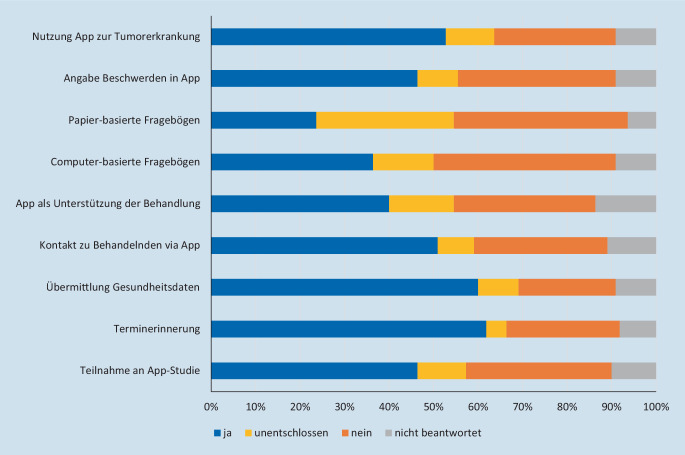
Balkendiagramm zur Darstellung der Patientenbefragung bezüglich der Akzeptanz von digitalen Apps in der Tumornachsorge

Im Rahmen der vorliegenden Studie wurden Fragen definiert, die speziell das Interesse der Patient*innen an der Anwendung von digitalen Apps im Rahmen der Tumornachsorge, z. B. zur Erfassung der PRO, eruieren sollten. Unabhängig von weiteren Faktoren wie Alter, Geschlecht, Schulabschluss usw. zeigte sich hier eine hohe generelle Akzeptanz (Nutzung einer App zur Tumorerkrankung: 58 %), wohingegen lediglich 30 % der Befragten nicht interessiert war. Auch bezüglich der Angabe von Beschwerden innerhalb einer App (51 %) sowie dem direkten Kontakt zu den behandelnden Ärzt*innen (57 %), der Übermittlung von Gesundheitsdaten in einer App (66 %) und der Terminerinnerung (67 %) wurde ein großes Interesse der Patient*innen gemessen (Abb. 1). Außerdem konnte nachgewiesen werden, dass sich nur 25 % der Patient*innen vorstellen könnten, regelmäßig papierbasierte Fragebögen zur Einschätzung der PRO zu beantworten, wohingegen diese Zahl bei computerbasierten Fragebögen auf 44 % anstieg.

**Abb. 2:**
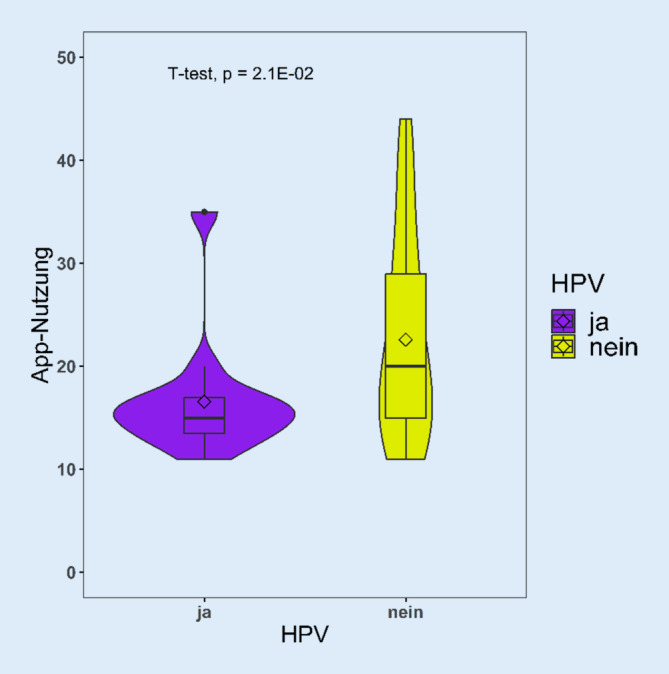
Violinen-Plot zur Darstellung der Mittelwerte aller Angaben zu den für die App-Nutzung relevanten Fragen zwischen für humanes Papillomavirus (HPV-)positiven und -negativen Patient*innen. Niedriger Gesamtwert: höheres Interesse an einer App-Nutzung

In einer weiteren Analyse untersuchten die Autoren, unter welchen Patient*innengruppen ein besonderes Interesse besteht bzw. ob gewisse Faktoren einen konkreten Einfluss darauf haben. Patient*innen unter 60 Jahren hatten ein signifikant höheres Interesse an einer App-Nutzung als Patienten über 60 Jahre (*p* = 0,0471; Tab. 1). Patient*innen ohne Smartphone gaben ein signifikant niedrigeres Interesse an (*p* < 0,00001; Tab. 1). Insgesamt fühlten sich 82 % der Befragten ausreichend über ihre Erkrankung informiert, dennoch gaben 55 % an, gern mehr Informationen über ihre Erkrankungen zu bekommen. Beide Gruppen zeigten ein signifikant höheres Interesse an einer App-Nutzung (*p* = 0,0053; *p* = 0,00014; Tab. 1). Weitere Faktoren wie Geschlecht, Tumorursprung, HPV-Assoziation, Behandlungsstatus und Betriebssystem des Smartphones waren nicht signifikant assoziiert. Die Frage nach der HPV-Assoziation wurde insgesamt allerdings auch am häufigsten nicht beantwortet. Bewertet man hier allerdings die Antworten zu allen Fragen, die auf das Interesse an einer App-Nutzung abzielen (s. Fragen in Abb. 1), unterschieden sich die HPV-positiven und -negativen Patient*innen signifikant (*p* = 0,021; Abb. 2). Das Alter unterschied sich im Mittelwert zwischen HPV-positiven (53,36) und HPV-negativen (59,15) nicht signifikant (*p* = 0,11).

Da sich in dieser Auswertung das Alter als einer der wichtigsten Einflussfaktoren herausstellte, wurde analysiert, ob sich ebenso in Bezug auf die weiteren Fragen nach dem Interesse an einer App-Nutzung ein signifikanter Unterschied ergibt.

**Abb. 3:**
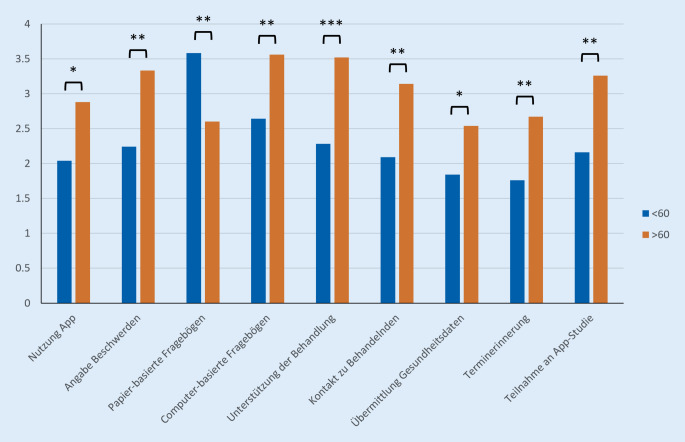
Säulendiagramm zur Darstellung der Angaben von < 60- und > 60-jährigen Patient*innen als Mittelwert zu den jeweiligen Fragen. Wert 1 bei der Auswahl von 1–5: großes Interesse, Wert 5: geringes Interesse. *:*p* < 0,05; **:*p* < 0,01; ***:*p* < 0,001

In allen Kategorien konnte hier ein signifikanter Unterschied zwischen den beiden Altersgruppen nachwiesen werden. Nur in der Frage nach papierbasierten Fragebögen zur Erfassung der PRO bestand ein signifikant höheres Interesse bei den über 60-jährigen Patient*innen (Abb. 3).

## Diskussion

Die onkologische Nachsorge von Tumorpatient*innen nach abgeschlossener Therapie gilt weiterhin als essenziell. Auch wenn die regelhafte Durchführung in klinischen Studien keinen signifikanten Überlebensvorteil ergab, ist dennoch die Detektion von Tumorrezidiven und metachronen Zweitkarzinomen ein wichtiges Ziel. Vor allem bei Kopf-Hals-Tumoren ist aufgrund der Schwere der Eingriffe und der posttherapeutischen Nebenwirkungen die Erhaltung bzw. Steigerung der Lebensqualität als ebenso wichtig anzusehen. Da die allermeisten Rezidive schnell zu verstärkten Beschwerden und damit einer Einschränkung der Lebensqualität führen, wird die Wichtigkeit der Erfassung der PRO deutlich.

Der Nutzen von PROM während der Tumorbehandlung konnte in einer randomisierten Studie am Memorial Sloan Kettering Cancer Institute in New York bei Patient*innen mit metastasierten Karzinomen bereits belegt werden. In 2 Studienarmen wurde entweder eine regelhafte Tumornachsorge bzw. klinische Untersuchung durchgeführt oder die Symptome der Patient*innen elektronisch überwacht („PRO-Gruppe“) [17]. Es zeigte sich sowohl eine signifikante Verlängerung des medianen Gesamtüberlebens und des qualitätsadjustierten Einjahresüberlebens in der „PRO-Gruppe“ als auch eine signifikante Reduzierung der notfallmäßigen Vorstellungen der Patient*innen [17]. Als möglicher Grund für das verlängerte Überleben wird eine frühere Reaktion auf Symptome und damit die Verhinderung von nachteiligen Konsequenzen für die Patient*innen diskutiert. Für die Erfassung von PRO in der Kopf-Hals-Onkologie stehen im deutschsprachigen Raum noch keine weit verbreiteten Tools zur Verfügung. In einzelnen Machbarkeitsstudien demonstrierten Zebralla et al. bezüglich Patientenzufriedenheit und Therapieadhärenz sowie individuellen Behandlungskonzepten allerdings gute Ergebnisse und eine hohe Patientenakzeptanz [18]. Eine Herausforderung ist grundsätzlich die Verknüpfung der Daten mit Krankenhausinformationssystemen (KIS) oder Praxisverwaltungssystemen (PVS) und die Portabilität der Daten bei Konsultationen in unterschiedlichen Kliniken oder Praxen.

Dennoch konnte in der vorliegenden Studie nachgewiesen werden, dass die Mehrzahl der Patienten Potenzial in der Entwicklung einer entsprechenden digitalen App als Ergänzung zur regelhaften onkologischen Nachsorge sieht. Hierbei ist explizit zu betonen, dass etwa 2 Drittel der Patienten mit einer Übermittlung von Gesundheitsdaten einverstanden wäre. Diese Ergebnisse zeigen sich etwas konträr zu den von Wald et al. 2022 erhobenen Daten [19]. Hier wurden im Vergleich zu der vorliegenden Studie sowohl Tumorpatient*innen der Hals-Nasen-Ohrenklinik des Universitätsklinikums Leipzig als auch Patient*innen ohne Tumorerkrankung ebenfalls bezüglich der Nutzung einer App zur Erfassung von ePROM befragt. Hauptsächlich wurde hierbei Wert gelegt auf den Vergleich zwischen Tumorpatient*innen und Patient*innen der allgemeinen Sprechstunde sowie zwischen Internet-Nutzern und solchen Patient*innen, die das Internet nicht regelhaft nutzen oder gar keinen Zugang haben. Die Mehrzahl der Tumorpatient*innen zeigte in dieser Studie kein Interesse an der Nutzung einer solchen App [19]. Im Fall einer Erfassung von ePROM sollten im Schnitt maximal 20 Fragen innerhalb von 10 min alle 3 Monate zu beantworten sein [19]. Die Autoren konnten in der vorliegenden Studie bestätigen, dass das Alter einen relevanten Faktor darstellt. Die in dieser Befragung statistisch signifikante Assoziation von HPV, bei der das Alter als mögliche Confoundervariable nicht ausgeschlossen werden kann, wurde in der Studie von Wald et al. nicht untersucht.

Neben der Erfassung von ePROM zur Symptomabfrage erhoffen sich eine Vielzahl an Patient*innen innerhalb der Befragung weitere Anwendungen in einer App, die den Alltag von Tumorpatient*innen erleichtern könnte. So wäre die Umsetzung eines Terminkalenders, der direkte Kontakt zwischen Patient*innen und behandelnden Ärzt*innen oder Angebote zu Sport und Ernährung bzw. logopädische Mitbetreuung möglich. Auch die Integration einer Patientenakte könnte bei wechselnden Kliniken und Praxen zu weniger Informationsverlust führen.

Sowohl diese Studie als auch die Etablierung einer digitalen App unterliegt gewissen Limitationen. Von angestrebten 200 Patient*innen konnten insgesamt nur 110 für die Studie rekrutiert werden. Zur Ablehnung der Teilnahme wurden unterschiedliche Gründe genannt (z. B. grundsätzliche Ablehnung zur Teilnahme an einer Studie, Desinteresse bei fehlendem Smartphone), wodurch im Erhebungszeitraum weniger Patient*innen zur Verfügung standen. Auch wenn die Mehrheit der Patient*innen ein Interesse an einer App hat, bleiben ein Drittel der Patienten, die darin keine Verbesserung der Behandlung sehen. Bezüglich der Entwicklung einer App steht bei dem Umgang mit sensiblen Gesundheitsdaten zwangsläufig der Datenschutz im Vordergrund. Des Weiteren besteht das Risiko einer Fokussierung der Patient*innen auf ihre Symptome, welche ggf. mit einer zusätzlichen psychischen Belastung einhergehen könnte.

## Fazit für die Praxis

- Die Erfassung von „patient-reported outcomes“ (PRO) während der Behandlung kann einen signifikanten Einfluss sowohl auf das Überleben als auch auf die Erhaltung bzw. Steigerung der Lebensqualität haben (bei Kopf-Hals-Tumoren hierzu bisher keine Daten).
- Die Erhaltung der Lebensqualität ist v. a. bei Kopf-Hals-Tumorpatient*innen aufgrund der posttherapeutischen funktionellen sowie ästhetischen Beeinträchtigungen im Alltag entscheidend.
- Kopf-Hals-Tumorpatient*innen waren in der vorliegenden Studie grundsätzlich aufgeschlossen gegenüber der Nutzung einer App, z. B. zur Erfassung der PRO, die im deutschsprachigen Raum bisher nicht flächendeckend zur Anwendung kommen, wobei das Alter aktuell noch ein relevanter Faktor ist.
- Die Entwicklung einer solchen App könnte die Patienten über einen Terminkalender, eine Patientenakte, eine Online-Sprechstunde, ein Sport- und Ernährungsangebot zusätzlich unterstützen und damit ein Bindeglied zwischen Arzt und Patient schaffen, sodass die Compliance im Rahmen der onkologischen Nachsorge erhöht wird.

## Supplementary Information

Fragebogen zur Erfassung des Interesses von Tumorpatient*innen an einer digitalisierten Form der Tumornachsorge.
