# Supplementary material for: Acceptance of digital apps in the follow-up of patients with head and neck tumors
Source: HNO. 2024 May 3;73(2):122–8. [Article in German] doi: 10.1007/s00106-024-01463-6 (PMC11772454; doi:10.1007/s00106-024-01463-6)
Supplement: Supplementary file 1 — Fragebogen zur Erfassung des Interesses von Tumorpatient*innen an einer digitalisierten Form der Tumornachsorge. [file 106_2024_1463_MOESM1_ESM.pdf]

Patient:innen - Fragebogen

Klinikum rechts der Isar

Technische Universität München

DIE DEUTSCHEN UNIVERSITÄTSKLINIKA®

Sehr geehrte Patientin, sehr geehrter Patient,

um unsere Behandlung weiter zu verbessern, benötigen wir Ihre Hilfe. Wir möchten gerne einschätzen, ob wir die Tumornachsorge und -therapie durch ein elektronisches Informationsangebot via einer Smartphone-Applikation (App) optimieren können. Mit Hilfe einer App könnten wir auch Ihre Beschwerden zu Hause erfassen und Sie möglicherweise frühzeitiger unterstützen.

Wir würden Sie daher bitten, uns folgende Fragen zu beantworten. Die Teilnahme ist anonym und dauert nur wenige Minuten.
Falls eine Frage für Sie aktuell nicht zutrifft, lassen Sie die entsprechenden Kästchen bitte frei.

Wir danken Ihnen sehr für Ihre Mithilfe!

Bitte kreuzen Sie unter/neben jeder Frage das Kästchen an, welches die Frage am besten beantwortet.

1. Geschlecht: ❑ weiblich ❑ männlich Alter: _______

2. Tumorursprung: ❑ Mundhöhle ❑ Oropharynx (u. a. Mandel, Zungengrund) ❑ Hypopharynx (tiefer Rachen) ❑ Kehlkopf ❑ Anderes

3. **Ist Ihr Tumor mit dem humanen Papilloma Virus (HPV) verbunden?**
   ❑ Ja ❑ nein ❑ nicht bekannt

4. **Was ist ihr höchster Bildungsabschluss**

   ❑ (noch) kein Schulabschluss ❑ Hauptschulabschluss ❑ Realschulabschluss
   ❑ Abitur ❑ Hochschulabschluss ❑ Promotion ❑ Anderer

5. **Welche Behandlung erhalten Sie aktuell bzw. ist geplant (Mehrfachauswahl möglich)**

   ❑ Operation ❑ Bestrahlung/Strahlenchemotherapie
   ❑ Systemtherapie (Chemotherapie, Immuntherapie) ❑ Keine Therapie/Nachsorge

6. **Fühlen Sie sich über Ihre Erkrankung ausreichend informiert?**
   (1 trifft voll und ganz zu – 5 trifft nicht zu)
   ❑ 1 ❑ 2 ❑ 3 ❑ 4 ❑ 5

7. **Wünschen Sie sich mehr Informationen zu Ihrer Erkrankung?**
   (1 Trifft voll und ganz zu – 5 trifft nicht zu)
   ❑ 1 ❑ 2 ❑ 3 ❑ 4 ❑ 5

8. **Wie würden Sie sich weitere Informationen über Ihre Erkrankung wünschen?**

   ❑ Informations-Broschüren ❑ Web-basiertes Patient:innenportal ❑ Smartphone-basiert
   ❑ Sprechstunde ❑ Selbsthilfegruppe ❑ Hotline für Tumorpatient:innen

9. Haben Sie ein Smartphone?

❑ Ja ❑ nein

10. Welches Betriebssystem nutzen Sie auf Ihrem Smartphone?

❑ IPhone OS (iOS) ❑ Android-basiert

11. Nutzen Sie Smartphone Applikationen (Apps)?

❑ Ja ❑ nein

12. Nutzen Sie das Internet, um Informationen über Ihre Erkrankung zu gewinnen?

❑ Ja ❑ nein

13. Würden Sie eine Smartphone App mit Informationen zu Ihrer Tumorerkrankung nutzen?

(1 trifft voll und ganz zu – 5 trifft nicht zu)

❑	❑	❑	❑	❑
1	2	3	4	5

14. Würden Sie Ihre Beschwerden bzw. Gesundheitsdaten in einer Smartphone App angeben?

(1 trifft voll und ganz zu – 5 trifft nicht zu)

❑	❑	❑	❑	❑
1	2	3	4	5

Wenn nein, warum nicht?

---

15. Würden Sie Papier-basierte Fragebögen zur Einschätzung Ihrer Beschwerden bevorzugen?

(1 Trifft voll und ganz zu - 5 trifft nicht zu)

❑	❑	❑	❑	❑
1	2	3	4	5

16. Würden Sie Computer-basierte Fragebögen (digitales Portal auf einer Homepage) zur Einschätzung Ihrer Beschwerden bevorzugen?

(1 Trifft voll und ganz zu - 5 trifft nicht zu)

❑	❑	❑	❑	❑
1	2	3	4	5

17. Könnte ihrer Meinung nach eine Smartphone App die Behandlung unterstützen?

(1 trifft voll und ganz zu – 5 trifft nicht zu)

❑	❑	❑	❑	❑
1	2	3	4	5

18. Würden Sie mit einer Smartphone App mit ihren Behandlern in Kontakt treten?

(1 trifft voll und ganz zu – 5 trifft nicht zu)

❑	❑	❑	❑	❑
1	2	3	4	5

19. Wären Sie einverstanden, wenn ihre Gesundheitsdaten durch eine Smartphone App an Ihre behandelten Ärzt:innen gesendet werden?

(1 trifft voll und ganz zu – 5 trifft nicht zu)

❑ 1 ❑ 2 ❑ 3 ❑ 4 ❑ 5

20. Wäre eine Erinnerung an Nachsorge-Termine durch eine Smartphone App hilfreich?

(1 trifft voll und ganz zu – 5 trifft nicht zu)

❑ 1 ❑ 2 ❑ 3 ❑ 4 ❑ 5

21. Könnten Sie sich vorstellen an einer Studie teilzunehmen bei der Ihre Tumornachsorge basierend auf Ihren Beschwerdeangaben in einer Smartphone App angepasst wird?

(1 trifft voll und ganz zu – 5 trifft nicht zu)

❑ 1 ❑ 2 ❑ 3 ❑ 4 ❑ 5

22. In welchen Bereichen der Behandlung und Nachsorge wäre eine Smartphone App für Sie am hilfreichsten (Mehrfachauswahl möglich)?

❑ Kontrolle der Beschwerden ❑ Informationsvermittlung ❑ Terminerinnerung

❑ Anleitung zu Therapien (Verhaltenstherapie, Entspannungstechniken, Bewegungstherapie)

❑ Kommunikation mit der Ärztin/Arzt

Welche Anregungen zur Verbesserung der Tumornachsorge und - behandlung haben Sie an uns?

____

____
